# Data privacy and smart home energy appliances: A stated choice experiment

**DOI:** 10.1016/j.heliyon.2023.e21448

**Published:** 2023-10-29

**Authors:** Hua Du, Qi Han, Dujuan Yang, Bauke de Vries, Thomas van Houten

**Affiliations:** Department of the Built Environment, Eindhoven University of Technology, 5600, MB, Eindhoven, the Netherlands

**Keywords:** Data privacy, Smart home appliances, Stated choice modelling, Privacy paradox

## Abstract

Data privacy in smart homes is receiving increasing attention due to the growing adoption of smart appliances. Adoption of smart appliances can bring benefits, including energy consumption reduction. This study investigates how people made the trade-offs between sharing privacy-sensitive data and the potential environmental and economic benefits of smart home energy appliances using discrete choice modeling. The findings reveal that the trade-off is mainly affected by four product attributes: the type of data that is processed, the reason why this data is processed, the data sharing frequency, and the financial benefit gained from the smart home appliances. Specifically, individuals tend to share less data daily for their daily routine convenience and demand a (theoretical) financial compensation for the data sharing. The results also show that privacy attitudes are not related to data sharing preferences, while socio-demographics, including gender, age, and income, are. The results emphasize the gap between people's attitudes and behaviors regarding data privacy. This research serves as a foundation for further investigations and can be used by smart appliance retailers, manufacturers, and governments for designing research and development focus and energy reduction incentives, respectively.

## 1 Introduction

The volume of data has grown exponentially with advanced data processing ability. Data processing is changing every individual's lifestyle, as wearables and smart devices are available to most people nowadays. Smart home appliances include smart washing machines, dishwashers, stoves, refrigerators, and many others. These appliances can be remotely monitored, accessed, or controlled and provide services based on users' needs [1,2]. There were over 1.2 billion smart home devices worldwide in 2018, which increased by approximately 45% compared with the year before [3].

The adoption of these appliances also contributes to energy reduction; therefore, smart homes are one of the EU's ten priority action areas in its Strategic Energy Technology Plan: Creating technologies and services for smart homes that provide smart solutions to energy consumers [4]. The rise of data processing entering our lives brings benefits and raises questions regarding personal data protection. On the one hand, Smart home appliances collect users' personal data to provide services and improve users' experiences. Companies are motivated to invest in data collection, processing, and storing to identify target groups, personalize their products and services, and create future business strategies. On the other hand, personal data includes individuals' financial information, medical information, energy consumption, habits, and locations, and there are potential risks of data loss and data corruption. Individuals may face much trouble, such as identity theft and credit card stealing, if these data fall into the wrong hands [5]. According to ForgeRock [6], 2.8 billion consumer data records were exposed to cybercriminals.

Individuals develop deeper concerns about the privacy and security of their personal information, and data privacy is more frequently discussed both in scientific research and mass media. Researchers are devoted to finding solutions for personal data protection [7,8]. ForgeRock [9] suggests that 90 % of people are concerned about identity theft and fraud, and 89 % are concerned that third parties can access personal data without consent. However, people do not act according to their privacy concerns and tend to have privacy-compromising behavior, which results in a dichotomy between privacy attitudes and actual behavior [10]. This inconsistency between privacy attitudes and privacy behavior is frequently referred to as the “privacy paradox” [11,12].

Nevertheless, new European privacy legislation named “General Data Protection Regulation (GDPR)” was implemented in May 2018, including data collection, usage, and storage requirements. Due to the GDRP, organizations have boundaries of data usage and sharing and increased responsibilities for data securing. The GDPR 2018 gives people the right to both consent and revoke consent. Moreover, the data controller must specify the processed data type, reasons for the processing, parties having access to the data, and the expiration date.

Smart home appliances are competitive in the market for their increased comfort, safety, reliability, and reduction in energy usage. It is necessary for real estate developers, smart home appliance manufacturers, and energy companies to invest in smart home technologies to attract customers. A deeper understanding of data privacy can make the difference between the success or failure of projects involving smart home appliances. Currently, most studies about privacy are focused on how individuals think about data privacy instead of how they act. This study aims to understand respondents’ trade-offs between data privacy and smart home appliance benefits, including energy efficiency.

## 2 Literature review

### 2.1 Smart home appliances adoption and energy consumption reduction

**Fig. 1:**
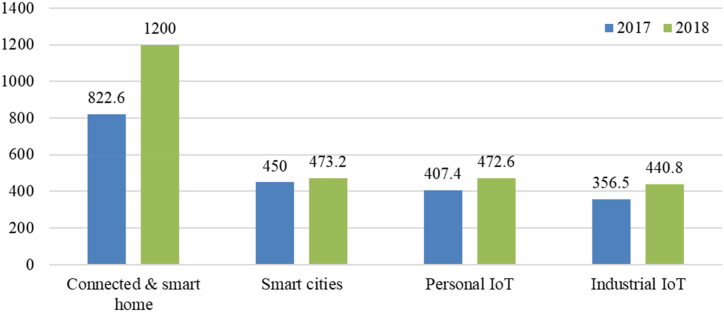
- Number of IoT devices worldwide in 2017 and 2018 [3].

Smart home appliances are usually the modern equivalents of existing products, and most appliances are capable of executing tasks that a human previously carried out. This is regularly named “home automation’ or “controllability’ [13]. Smart home appliances are becoming popular (see Fig. 1) due to these “controllability’ benefits and providing comfort, safety, health monitoring, and reliability. Marikyan et al. categorized three comfort types of smart home appliances: automation of daily routines, remote home management, and acting according to the detected environment [14]. For example, smart thermostats allow users to turn on the heat or air conditioning before arriving home [15]. These appliances also have interoperability, which allows them to use data from different smart appliances and work together for optimized service [16]. The adoption of smart home appliances is influenced by several factors, including convenience and connectedness, perceived benefits, and trust in the manufacturer as well as concerns regarding device privacy risks [17,18]. Among these factors, the primary driver for the adoption of smart home appliances is the desire for convenience and connectedness [17].

Besides, Smart home appliances contribute to reducing energy consumption to a large extent. For example [19], shows that 5 to 15% of energy reductions can be achieved with direct feedback options from smart appliances. Furthermore [20], implies that the more direct the feedback is, the higher the potential savings are [21]. suggest that the investment in smart home appliances reduces energy due to the improved coordination between electricity, thermal, and gas grids [22]. summarize that smart home appliances are designed to achieve energy reduction with three approaches: 1) the product provides information to the household; 2) the product enables the household to control their smart home; 3) the product controls the house on behalf of the household.

### 2.2 Data privacy theory and link with smart homes

Smart home appliances need to gather, analyze, and use various data to achieve the aforementioned benefits. For example, four categories of data will be needed for smart homes to achieve energy consumption reduction [1]: (1) Daily routines to discover an individual's behavior patterns; (2) Identity of a person to activate personal preferences; (3) The current location of an individual to determine someone's location; (4) Households' activities to predict and detect (unusual) behavior and anticipate when necessary. Hence, households' choices of smart home appliances are also influenced by their attitudes toward data privacy. Privacy concerns have been identified as one of the major barriers to smart home appliance adoption [18].

More specifically, two types of trade-offs influence an individual's privacy behavior: the privacy calculus trade-off (i.e., the trade-off between expected benefits and privacy risks) and the risk calculus trade-off (i.e., the trade-off between privacy risks and the efficacy of coping mechanisms) [23]. identified 15 privacy theories, and one of the most discussed theories is utility maximization. Utility maximization suggests that an individual makes a risk-benefit analysis where the negative consequences are rationally weighed against outcomes, and people aim to minimize information disclosure risks and maximize potential benefits [10]. Some studies use the perception of risks, trust, and interest to determine an individual's willingness to provide personal information [24]. Moreover [25], found a trend of using privacy concerns as the central construct to measure privacy risks.

**Fig. 2:**
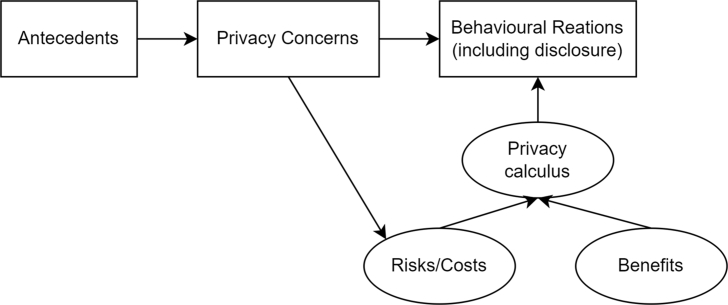
– APCO model by Potoglou et al. [27].

Several studies suggest that socio-demographics, personality, past experiences, and privacy awareness factors are antecedents of privacy concerns. For example, Lee et al. [26] found that gender, age, education, and income significantly influence privacy concerns. Potoglou et al. [27] proposed the APCO model, which demonstrates the influence of privacy concerns and privacy calculus on privacy-related behaviors, as depicted in Fig. 2. The model provides insights into how various antecedents, including demographic characteristics, can impact an individual's privacy concerns.

However, researchers also found a contradiction between how individuals think about privacy (i.e., concerns, trust, and interest) and how individuals act the privacy paradox. Research settings for studying the privacy paradox fall into two categories: social and transactional situations. Social situations mostly explain the paradox concerning social network sites. In contrast, the transactional situation is often used in multiple contexts like e-commerce, smartphone usage, and online shopping [11].

A survey with Likert scale questions can evaluate individuals’ attitudes, but it cannot measure their behaviors or intentions. Furthermore, according to Ref. [11], self-reports are unreliable to measure behavior accurately. One possible way of studying behavior is by conducting behavioral experiments in real situations. For example [2], tested actual behavior by letting test residents move into a dwelling and track their energy consumption. However, these experiments are not frequently used since it is costly and time-consuming. An alternative is to use the stated choice experiments. For example [28], implemented a stated preference experiment to test the preferences for various privacy settings in the context of security and surveillance of train/metro facilities in Europe [5,27]. used stated preference experiments to test the behavioral intention concerning internet surveillance and e-commerce.

In general, most studies regarding privacy focused on how individuals think about data privacy instead of how they act. Moreover, most studies studied data privacy in online shopping and social media contexts. This study aims to study the trade-off between data privacy and energy consumption reduction benefits in smart home appliances. A discrete choice experiment was developed to investigate the trade-off between sharing privacy-sensitive data and obtaining the benefits of smart home appliances, such as energy efficiency.

## 3 Methodology

### 3.1 Discrete choice modeling

A Discrete Choice Experiment (DCE) is used to investigate the trade-offs between data privacy and the benefits of smart home appliances. The discrete choice modeling is based on the random utility theory (RUT), which suggests that individuals strive to maximize total utility or satisfaction from consuming goods or services [29]. In DCE, the alternatives are decomposed into different attributes, and the value of the alternatives and attributes respondents perceived can be measured [30]. The utility (*U*_*nj*_) that individual *n* perceives from alternative *j* can be partitioned into two separate components, an observed component (*V*_*nj*_) and an unobserved component (*ε*_*nj*_), such that:

(1) $$U_{nj}=V_{nj}+\varepsilon_{nj}$$

The utility is calculated by adding the observed component as the unobserved component (*ε*_*nj*_) is a stochastic error component. The observed utility is the sum of the weight of attribute (*β*_*j*_) multiplied by the score of alternative *j* on attribute *k* of individual *n*, as shown in equation (2).

(2) $$V_{nj}=\sum_{k=1}^{K}\beta_{k}x_{njk}$$

Both the multinomial logit model (MNL) and mixed logit model (ML) are presented in this study. Compared with MNL models, ML models assume that there is a taste variation among the respondents. ML models consider taste heterogeneity by estimating the standard deviation of the attribute parameters. They account for the correlations across an individual's choice by estimating all sequences of choices made by one respondent [31]. In general, a higher number of repetitions in ML will result in a higher accuracy of the results and a higher explanation power (stronger utility scores) [32]. state that this model is currently the most promising state-of-the-art discrete choice model. Halton draw was used in the ML model [31]. suggests that Halton draws provide better results than random draws for mixed logit estimations, but it is unclear what number of draws is best [33]. concluded that 125 draws provided the best balance between results and processing time, which is adopted in this study.

Two tests are adopted to evaluate the model performance: the McFadden Rho Squared Test and the Adjusted Rho Squared Test. Both tests use log-likelihood values to evaluate the model performance. The total fit of the model can be determined by McFadden's R^2^. According to Ref. [34], values for R^2^ between 0.2 and 0.4 represent sufficient goodness of fit. Values higher than 0.5 are considered unrealistic for behavioral experiments. The adjusted Rho Square takes into account the degrees of freedom and the number of respondents (k). Thus, it can be discovered if a model scores a higher Rho2 because it is the superior model or has a higher number of predictors (independent variables). The adjusted Rho2 is, therefore, essential when sub-models are being used.

### 3.2 Survey design

The experimental design framework of [32] is used. The choice experiment consists of multiple “choice settings' per observation and one choice set consists of three alternatives. One of these alternatives in each choice set is the “no preference’ option. The two other alternatives contain a finite number of attributes. An attribute has several levels that may vary per alternative.

The survey contains two parts: the first part includes social-demographic questions and perceptions on data privacy and energy conservation; the second is the stated choice experiment. Socio-demographic variables included in this study are age, gender, current occupation, household composition, gross income, and highest finished education. The Dutch census called “Centraal Bureau voor de Statistieken” (CBS) is consulted for developing correct measurement levels.

#### 3.2.1 Perception of privacy concerns and energy conservation

Statements used for evaluating individuals' perceptions of privacy concerns and energy consumption are shown in Table 1. According to the privacy theory, individuals are willing to sacrifice their privacy if a positive trade-off is available [35]. In the privacy calculus model developed by Ref. [24], the perceived risk is based on trust and concerns. The model focuses on internet use and may not be useful in this study. However, questions in Dinev & Hart's research that indicate the perception of privacy concerns can be modified to fit the purpose of this research [26]. proposed a similar modification approach: respondents' opinions about energy consumption are on the other end of the scale as the trade-off is between data privacy and energy consumption. Factors affecting energy-saving behaviors are adopted from Ref. [36], which includes three questions about energy consumption and two questions regarding general opinion on sustainability. The 5-point Likert scale from “Strongly Disagree’ to “Strongly Agree’ is used to measure the aforementioned questions and factors.

**Table 1:** Perception of privacy concerns and energy consumption.

Statements regarding data privacy	
a	I'm concerned about third parties being able to access my personal data
b	I'm concerned that parties are not keeping my personal information secure
c	I'm concerned that the information I submit on the internet could be misused
d	I'm concerned about parties building a profile of me to predict my consumer behavior
e	I'm concerned that I have insufficient control over the data that is collected about me
Statements about perception of energy conservation	
a	It is important to me to reduce my energy consumption
b	I'm interested in having better insight into my energy consumption
c	I'm interested in smart technology that helps me reduce energy consumption
d	I'm interested in the latest technology and gadgets
e	I'm concerned about the environmental effects of Global Warming

#### 3.2.2 Discrete choice experiment

Individuals' behavioral intentions can be measured through the risk-benefit analysis. In the stated choice experiment, respondents weigh the risks and benefits of alternatives and make decisions. Six attributes are included in the experiment. The attributes and levels are shown in Table 2. One level is assigned as the “reference level’ for every attribute. This level is best relatable to the current situation and will be used in the survey's introduction to inform the respondent about the current situation.

**Table 2:** Experimental design attribute and level identification.

Attribute	Attribute levels
What type of data is processed?	1. Sensor data of your total energy consumption *[Reference Level]* 2. Sensor data of your total energy consumption + Specified for every individual electronic product 3. Sensor data of your total energy consumption + Specified for every individual electronic product + Personal data collected from smart home appliances 4. Sensor data of your total energy consumption + Specified for all electronic products in your house + Personal data collected from smart home appliances + Real-time GPS location data of household members
Why are such data processed?	1. To inform you about the energy usage of products in your house *[Reference Level]* (Example: Inform about the energy usage in your living room) 2. To remotely manage the products in your house. (Example: Manage the room temperature from a distance) 3. To control daily routines in your house (Example: Control the dishwasher so that it turns itself on when energy usage is low) 4. To automate smart home appliances that detect and act (Example: Automate so that lights are turned off when you leave the room)
Who has access to your data?	1. Energy Provider *[Reference Level]* 2. Technology company
When will processing take place?	1. Every Month *[Reference Level]* 2. Every Day 3. Every Hour 4. Every Minute
When will data be removed?	1. After 10 days *[Reference Level]* 2. After 1 Month 3. After 1 year 4. After the product is out of use
Trade-off	1. Environmental benefit and no financial benefit *[Reference Level]* 2. Environmental benefit and financial benefit of € 5 per month 3. Environmental benefit and financial benefit of € 10 per month 4. Environmental benefit and financial benefit of € 15 per month

The data processing types in different smart home appliances vary, and an abundant number of data types can be named. The data can be set by the user and detected by the product. The data type is not enlisted, such as time, temperature, or energy consumption, to avoid any misunderstanding. The first two levels focus on energy consumption data, while the last two levels include household members’ data processed by the smart home appliance.

The next attribute is the reasons for processing the data. The data processed by smart appliances can benefit both the users and the data controller and processor. In this research, only users' benefits, namely appliances’ functions, are considered. The levels include informing, managing, controlling, and automating energy consumption, and the respondent benefits from letting the technology take additional responsibilities with every next level. An example for each level is provided to help respondents understand.

Two actors having access to the data consist of the “Act’ attribute. Energy providers use data to optimize their energy distribution and for billing purposes. For technology companies, the data can optimize their products and services. Their ultimate goal of data processing is to increase their profit on these services. The attribute of data frequency sharing includes four levels. More frequent data sharing relates to a higher frequency of data processing of smart appliances, which also suggests that these appliances are more capable of adapting to consumers’ demands. More frequent data sharing can also accelerate product energy efficiency improvement.

When processing has taken place, the data controller typically stores consumer data in their own databases [37]. With the new regulations of the GDPR, the user has the right to revoke data storage at all times. Smart home appliances cannot perform more optimally when data is unavailable optimally. Contrarily, smart home appliances have an increased vulnerability to data breaches since they contain large quantities of consumer information. There are risks of personal data leaks since smart home appliances are mostly connected to the internet. Data removal can impact choice behavior if individuals have concerns regarding data privacy. The retention times are set after 10 days, 1 month, 1 year, and after the product is out of use.

The “trade” attribute tests respondents’ trade-off between data collection in smart home appliances and environmental benefits and financial compensations. The actual potential compensations for respondents depend on the size of the dwelling, household composition, income, energy prices, and other possible influences and vary from person to person. Thus, the financial compensation in this attribute is based on potential incentives, not on market prizes and energy reductions. Nevertheless, the hypothetical compensations must be realistic and reasonable from a cognitive perspective [38]. The reference level of this attribute only includes environmental benefits. Extra financial compensations of €5,- €10,- and €15 are added in the other three levels. These values are well-rounded and related to the values that are mentioned in the other studies [39,40].

There are five 4-level attributes and one 2-level attribute in the experiment design. The full factorial design includes 2048 alternatives and is too large to test. Instead, a fractional design of 32 alternatives will be used. Statistical Analysis Software (SAS) was used to calculate the optimal design. There will be 16 choice sets, and each choice set includes two alternatives. The flagging approach in SAS was used to make sure that the levels are not similar in a choice set. The goodness of the experiment design is evaluated. The relative D-efficiency is 55.62 on a 0 to 100 scale (a score of 100 suggests that the design is balanced and orthogonal, while 0 suggests that one or more levels cannot be estimated). In this case, the experiment design is acceptable for two reasons: a 56 score can be considered an average result [41], and the covariance matrix did not reveal significant errors. Effect Coding was adopted for allowing non-linear effects in the different levels of the attributes. Effect coding for 2-level and 4-level attributes is shown in Table 3.

**Table 3:** Effect coding and utility.

Number of levels	Level	β_1_	β_2_	β_3_	utility
2	Level 1	1			$\beta_{1}*1$
	Level 2	−1			$\beta_{1}*\left(-1\right)$
4	Level 1	1	0	0	$\beta_{1}*1+\beta_{2}*0+\beta_{3}*0$
	Level 2	0	1	0	$\beta_{1}*0+\beta_{2}*1+\beta_{3}*0$
	Level 3	0	0	1	$\beta_{1}*0+\beta_{2}*0+\beta_{3}*1$
	Level 4	−1	−1	−1	$\beta_{1}*\left(-1\right)+\beta_{2}*\left(-1\right)+\beta_{3}*\left(-1\right)$

Each respondent answered 8 out of 16 choice sets (questions) in LimeSurvey, resulting in no bias in choice order. Consequently, there might be a discrepancy in the survey results since some choice settings can appear in more surveys. This issue will be solved prior to the closure of the survey by manually composing the last couple of surveys. The survey was tested before its activation. The test panel identified two problems. First, the survey was unsuitable for the execution on a mobile phone. An unnecessary amount of scrolling was required, which resulted in uncareful reading. The survey was improved and made primarily suitable for a mobile phone. Second, several words were considered complicated to understand. The issue was solved by expanding the explanation in the introduction.

## 4 Results and discussion

Two hundred fifty-six full discrete choice surveys were acquired after excluding the unfinished surveys and a survey finished in only 48 s. A small sample was pre-tested for an initial belief of the parameter values for sample size calculation, using the method introduced by Ref. [42]. The calculations predicted that several parameters are insignificant even with a considerable sample size (>1000). The remaining parameters were significantly below the indicated 125 surveys, indicating that the sample size of 250 respondents will be sufficient. The survey was implemented in June 2019.

The socio-demographics of the respondents are shown in Table 4. It is found that the elderly are underrepresented in this study. 19.2 % of citizens fall in the group of age more than 65 at the national level, but only 1.6 % of the respondents belong to this group.

**Table 4:** Descriptive statistics of socio-demographic characteristics.

Socio-Demographic Characteristics	Levels	Sample Count	Sample Percentage
Age	Age <19	22	8.5
	19∼29	78	30.0
	30∼45	56	22.9
	46∼65	96	37.5
	Age >65	4	1.6
Gender	Men	130	50.8
	Women	126	49.2
Occupancy	Student	58	22.6
	Employed (Full time)	142	55.5
	Employed (Part-time)	44	17.2
	Unemployed	8	3.1
	Retired	4	1.6
Household Composition	Single Person	39	15.3
	Two Person	77	30.2
	Family with children	134	52.5
	Single Parent	6	2.0
Income	< €20.000	63	24.6
	€20.000 - €30.000	26	10.2
	€30.000 - €40.000	44	17.2
	€40.000 - €50.000	27	10.5
	> €50.000	67	26.2
	Rather not say	29	11.3
Highest finished education	Secondary Education (VMBO)	9	3.5
	Secondary Education (HAVO, VWO)	30	11.7
	MBO	62	24.2
	HBO	86	33.6
	University (Bachelor)	37	14.5
	University (Masters)	32	12.5

### 4.1 Statement analysis

The Cronbach's Alpha (α) was used for testing statements' reliability. The test result for the five privacy statements is 0.86, higher than the recommended reliable level of 0.70. It implies a relatively high inter-correlation among the privacy statements. Thus, all five statements (privacy statements) were used for further analysis. The Cronbach's Alpha (α) for the energy consumption statements is 0.64, lower than the recommended level (0.70). The test results were 0.78 after removing statements 4 and 5. Test results suggest a relatively high intercorrelation among only statements 1, 2, and 3, but not among statements 4 and 5. Hence, only statements 1, 2, and 3 (energy consumption statements) were used for further analyses.

The ANOVA analysis was used to test the correlation between socio-demographic factors and statements. Previous studies have argued that women tend to feel more concerned about their privacy than men [26]. Results in this study partly confirmed this hypothesis as all mean values are higher and the standard deviations are lower for women. Moreover, test results show that statements 3 (misuse of data) and 5 (control over data) are significant at the 0.05 level. Test results also suggest a correlation between age and perception of privacy concerns for statements 1 and 2 (significant at 0.01). Statement 1 and 2 are regarding third-party access and data security, respectively. In general, older respondents have more concerns about their privacy than younger respondents, which is also found by Ref. [26]. Statement 1 and 2 are also correlated with income at 0.01 significant levels, and higher-income groups have greater privacy concerns.

The ANOVA analysis for the three energy consumption statements and age variable shows that all statements are correlated with the age variable at significant levels higher than 0.10. Older respondents attach more importance to energy reduction and are more interested in energy technology and having consumption overviews. Test results also show that men are more interested in technologies helping reduce energy consumption than women, at the significant level of 0.10.

### 4.2 MNL and ML model estimation

The discrete choice experiment was analyzed in Nlogit. The estimations, including coefficients, standard error, and significance of the MNL model and ML model, are shown in Table 5.

**Table 5:** MNL and ML model estimation.

Parameters	MNL model		ML model	
	Coefficient	t-value	Coefficient	Std. Dev
Constant	−1.501***	−18.37	−2.606***	2.147***
Type 1: Share sensor data	0.401***	7.09	0.612***	0.674***
Type 2 + Data specified per product	0.308***	5.66	0.450***	0.227*
Type 3 + Data collected by Home Appliance	−0.021	−0.37	−0.015	0.367***
Type 4 + GPS data household members	−0.688	–	−1.047	–
Why 1: Inform user of energy usage	−0.056	−0.93	0.009	0.477***
Why 2: Remotely manage energy consumption	−0.202 ***	3.32	−0.230***	0.291***
Why 3: Remotely control daily routines	0.200 ***	3.52	0.199**	0.232*
Why 4: Automate Smart Home Appliances	0.056	–	−0.001	–
Act 1: Energy Companies	0.055	−0.09	−0.001	0.627***
Act 2: Technology Companies	−0.055	–	0.001	–
Share 1: Every Month	−0.034	−0.64	−0.015	0.158
Share 2: Every Day	0.184 ***	3.30	0.28***	0.314***
Share 3: Every Hour	−0.055	−0.95	−0.851	0.330**
Share 4: Every Minute	−0.095	–	0.579	–
Remove 1: After 10 days	0.086	1.28	0.125	0.280**
Remove 2: After 1 month	0.054	0.88	0.119	0.295**
Remove 3: After 1 year	−0.084	−1.42	−0.095	0.091
Remove 4: After the product is out of use	−0.056	–	−0.149	
Trade 1: Environmental benefit	−0.440***	7.65	−0.685***	0.724***
Trade 2: Environmental benefit + €5	−0.053	−0.94	−0.079	0.219*
Trade 3: Environmental benefit + €10	0.121**	2.09	0.183 **	0.070
Trade 4: Environmental benefit + €15	0.372	–	0.581	
Rho-square	0.081		0.268	
Adjusted Rho-square	0.075		0.262	

Significance codes: (0.01 = “***”) (0.05 = “**”) (0.1 = “*”).

The MNL model and ML model results are shown in the middle and the right column in Table 5, respectively. As shown in Table 5, the attributes' significance does not change much in the two models. However, there is a difference in the performance of the MNL model and the ML model. The MNL model scores a McFadden Rho^2^ of 0.081 and an adjusted Rho^2^ of 0.075, while the McFadden's Rho^2^ and adjusted Rho^2^ for the ML model are 0.268 and 0.262. A value between 0.2 and 0.4 is considered an excellent fit for choice experiments [29]. The mixed model considers panel data, which means respondents persisted unobserved factors are considered. To be more specific, individuals' different preferences are considered in the ML model by assuming that all parameters converge to distribution across the population.

In the ML model, all attributes show significance in standard deviation. The standard deviation indicates to what extent respondents have similar results. A small standard deviation suggests that the respondents have similar results and vice versa. The results in the last column of Table 5 indicate that there are taste differences toward every attribute within the population.

The utility value should be interpreted by comparing the value among attributes and levels, and a greater utility value suggests more preference than a smaller utility value. The sum of the least attractive utility values in the ML model is −2.963, while the constant parameter is −2.606. It shows that all choice situations are experienced as more attractive than the “no preference” option in the ML model. Thus, the respondents are willing to choose a smart home appliance, indicating that respondents are not against implementing smart appliances in their homes. Only two attributes do not show any coefficient significance in the ML model: Act and Share.

The findings regarding the attribute “Type” indicate that respondents are generally willing to share energy usage data but are reluctant to share personal data, particularly GPS data. The disparity between the two extreme levels (1 and 4) within this attribute is substantial, highlighting the significant influence of data type on overall utility. Similar patterns are observed in the results for the attribute “Why” in both the ML and MNL models. The majority of respondents prefer the third level, indicating that respondents prefer to control smart home appliances remotely, while people will not adopt smart home appliances for managing energy consumption. The impact of data sharing frequency reveals that sharing data on a daily basis has a significant positive effect on respondents’ choices. Additionally, respondents express a preference for data to be removed as quickly as possible. Regarding the attribute “Environmental Benefit,” the results indicate that as the financial benefit increases, the utility perceived by respondents also increases. Furthermore, respondents exhibit a clear dislike toward the level where no financial benefit is provided.

### 4.3 Estimation results with interaction

Different interaction variables are tested in the ML model to include the individual-specific parameters in the models, which is suggested as the most accepted approach to include demographics [32]. It was decided to focus on the interaction between the personal characteristics and the attributes Type and Trade. The choice is based on three reasons: 1) the attributes are best representing the trade-off between data and the benefits of smart home appliances; 2) they are both homogeneous according to the mixed logit model; 3) The utilities of the attribute levels vary to a greater extent compared with other attributes. Tested socio-demographic variables are gender, age, income, and education, as these variables have a significant correlation with the perception of privacy concerns, as introduced in section 4.1. Categories of the age, income, and education variables were merged for further analysis. The number of categories for variable age is reduced from five to three by merging the first two and last two categories, while the income categories have been reduced from six to four by merging the second, third, and fourth categories. Lastly, the categories for the education variable were reduced from six to four by merging categories one and two and categories five and six.

All models are executed twice by changing the coding for the social-demographic variable to evaluate the significance of interactions between the attribute levels and the demographic variables. The results for the interaction terms are shown in Table 6 (the education variable is not included as there is no significance). The ML model with assigning random variables to attributes Why, Act, Share and Remove and no variable interaction (base model) was simulated to compare model performance. The interaction terms are added to this base ML model instead of the ML model from section 4.2. The Rho-square and Adjusted Rho-square for this base ML model are 0.257 and 0.252, respectively.

**Table 6:** Interaction terms between Type, Trade and the socio-demographic variables.

Interaction	Male	Female	Age <29	Age 30–45	Age >46	Income < € 20k	Income € 20-50k	Income > € 50k	Rather not say
Type 1	−0.275***	0.275***	0.031	0.300***	−0.331***	0.094	0.283***	−0.166*	−0.211
Type 2	−0.026	0.026	−0.016	0.017	−0.001	0.134	0.002	0.022	−0.158
Type 3	0.008	−0.008	0.087	−0.056	−0.031	0.010	−0.066	−0.088	0.144
Type 4	0.293	−0.293	−0.102	−0.261	0.363	−0.238	−0.219	0.232	0.225
Rho-square	0.264		0.263			0.262			
Adjusted Rho-square	0.259		0.257			0.255			
Trade 1	−0.038	0.038	−0.135	−0.015	0.150	−0.069	0.069	0.092	−0.092
Trade 2	−0.027	0.027	0.081	−0.179*	0.098	0.061	−0.011	0.077	−0.127
Trade 3	−0.047	0.047	0.035	0.137	−0.172**	0.073	0.013	−0.059	−0.027
Trade 4	0.112	−0.112	0.019	0.057	−0.076	−0.065	−0.071	−0.11	0.246
Rho-square	0.263		0.258			0.258			
Adjusted Rho-square	0.257		0.252			0.252			

The performances of the ML model with interaction terms and base ML are compared. The comparison shows that adding interaction terms increased the model performance differently. The ML model with the interaction of attribute “Type” and gender has the best performance with Rho-square of 0.264, while the ML models with the lowest Rho-square (0.258) are with interaction terms of Trade-age and Trade-income.

The utility values for all fourth-level options in Table 6 are manually calculated (as the sum of the first three levels), and the significance of these interactions is not tested. The results indicate that women are significantly more willing to share sensor data compared to men. Regarding age groups, respondents aged between 30 and 45 exhibit a significant preference for sharing less data, whereas respondents over the age of 46 show a greater willingness to share more data. The interaction between age and the “Trade” attribute reveals that individuals aged 30 to 45 prefer a financial benefit level of €5, while those older than 45 prefer a €10 financial benefit level. Furthermore, contrasting preferences are observed between the middle-income group (€20-50k) and highest-income group (>€50k) in terms of data sharing. The former group prefers to share less data, while the latter group tends to share more data. Interestingly, the model results indicate that individuals’ preferences for financial benefits are not correlated with their income level.

The study investigated the interaction between privacy and energy conservation attitudes with the “Type” and “Trade” attributes. Privacy and energy conservation attitudes are measured with five statements, respectively, as described in section 3.2, which is downsized using the principal component analysis for interaction. The results of the principal component analysis are shown in Appendix A. The first component was used for the interaction, and the results of the interactions are shown in Table 7. The interaction of privacy concerns and energy conservation attitudes improve the performances of the ML model to a very limited extent. Specifically, the interaction terms of privacy attitudes do not show any significance as shown in Tables 7 and it is not even related to their preference of sharing different types of data. The factorized energy conservation attitudes display significance only in their interaction with the first levels of the “Type” and “Trade” attributes. Respondents with more positive energy conservation attitudes exhibit a greater inclination to choose options involving the sharing of lesser data and appliances that lack economic benefits.

**Table 7:** Interaction variables with privacy and energy conservation attitudes.

Interaction between	Type1	Type2	Type3	Type4	Rho-square	Adjusted Rho-square
Privacy	0.006	−0.045	−0.031	0.07	0.258	0.252
Energy Conservation	0.130**	0.012	0.003	−0.145	0.259	0.253
	Trade1	Trade2	Trade3	Trade4		
Privacy	−0.064	0.021	−0.010	0.053	0.258	0.252
Energy Conservation	0.151**	−0.016	−0.011	−0.124	0.259	0.253

### 4.4 Discussion and result implication

The trade-off between adopting smart home appliances for energy reduction and privacy is primarily determined by four key attributes: the type of data being processed, the purpose of data processing, the frequency of data sharing, and the financial benefits. Generally, respondents demonstrate a willingness to share sensor data related to energy consumption while being more reluctant to share personal GPS data. This suggests that the majority of respondents exhibit privacy-protective behavior. Additionally, respondents display a preference for appliances that assist them in their daily routines and require daily data sharing. This indicates that most individuals choose smart home appliances based on their usability. Furthermore, respondents’ behavioral intentions are influenced by the perceived financial benefits associated with the use of smart home appliances.

This study found that women, older individuals, higher-educated individuals, and high-income individuals generally have more concerns about their data privacy, which aligns with previous studies [27,43,44]. For instance, Sheehan [43] observed that high-income individuals have more privacy concerns. However, the privacy paradox [11,12] suggests that having greater privacy concerns does not necessarily translate into adopting privacy-protective behaviors. This study examines the degree of individuals' concerns and differences in choice behavior to explore privacy protection behavior further. The privacy paradox is apparent when looking at the interaction with socio-demographic variables and choice behavior. For example, the education variable shows significant interactions with privacy concerns. However, it does not significantly influence the behavioral intentions of individuals. This phenomenon is also shown through the age and income variables. Only the gender variable shows consistency as women show not only concerns regarding data privacy but also show more privacy-protection behavior. Overall, not all the socio-demographics impacting individuals' privacy concerns also impact their privacy protection behavior. It is also found that the attitudes towards privacy do not affect respondents’ preference regarding the type of data shared and the economic benefit gained. However, it is found that people with more positive energy conservation attitudes are less likely to seek economic benefits. These findings suggest that while increasing awareness about privacy may not directly impact the trade-off, raising awareness about energy conservation has the potential to encourage energy-saving behaviors through educational and informational campaigns.

The following implications can be derived from the results obtained in relation to the trade-off involved in selecting smart home appliances, individuals' preferences regarding privacy-related behaviors, and the correlation between attitudes and preferences. First, the adoption of smart home appliances can be encouraged by providing information regarding living assisting functions and economic benefits due to better energy management or appliances' energy-efficient features. Smart home appliance retailers can emphasize these product features as the selling point. Secondly, manufacturers of smart home appliances can focus on balancing using less data and achieving optimal usage for successful businesses. The government can put effort into encouraging manufacturers to provide more energy-efficient products and provide information campaigns and education activities to increase people's energy conservation awareness.

## 5 Conclusion

Smart home appliances include smart washing machines, dishwashers, thermostats, refrigerators, and others that can be remotely monitored, accessed, or controlled and provide services that respond to users’ needs. One of the essential benefits of smart home appliances is the potential for energy reduction. The energy reduction is achieved by both smart appliances themselves and (in)direct feedback options to the user. Personal data are essential for providing such options for making energy-efficient decisions. However, the more personal data are processed, the larger the potential privacy consequences are when these data are abused. The trade-off between storing personal data on appliances connected to the internet and the benefits of adopting smart home appliances was investigated in this study through discrete choice modeling, and this research aimed to provide more insight into the choice behavior of individuals in the context of a smart home.

Study results show that the trade-off is mainly determined by four attributes: the type of data that is processed, the reason why this data is processed, the data sharing frequency, and the financial benefit that the smart home appliances can obtain. Besides, people's age, income, and energy conservation attitudes impact their tastes regarding Type and Trade attributes. It was found that there is a clear difference between what individuals claim about data privacy and how they behave. The confirmation of the privacy paradox suggests that privacy behaviors cannot be measured based on attitudes. However, people show more consistency between energy conservation attitude and behavior, as people with more responsible energy attitudes are less likely to seek financial benefits.

This research is a foundation for more (privacy-specific) research. In this study, theoretical smart home appliances were surveyed, which can confuse some respondents during the survey, as it is unknown whether individuals show different behavior when they are more familiar with these products, which is due to the hypothetical nature of the stated choice experiment. Another limitation is that the elderly group is underrepresented, as discussed in section 4. Future research should consider the privacy trade-off for realistic products. Moreover, this research mainly focused on the trade-off between data and financial benefits. The benefits are theoretical and, therefore, unrepresentative when testing actual existing products. Future research can consider additional benefits such as comfort, safety, health, and reliability. Another path is to investigate appliances with different privacy-sensitive levels. For example, applying smart home appliances in the bedroom might be considered more privacy-invasive than implementing a smart washing machine in the garage.

## Ethics statement

The data for this study was collected in June 2019, prior to the mandatory requirement of ethics approval in the university (2020).

## Data availability statement

Data will be made available on request.

## CRediT authorship contribution statement

**Hua Du:** Formal analysis, Writing – original draft. **Qi Han:** Conceptualization, Writing – review & editing. **Dujuan Yang:** Writing – review & editing, Conceptualization. **Bauke de Vries:** Conceptualization, Writing – review & editing. **Thomas van Houten:** Conceptualization, Data curation.

## Declaration of competing interest

The authors declare no conflict of interest in preparing this paper.

## Figures and Tables

**Table A:** Principle Component Analysis

Privacy statements					
Kaiser-Meyer-Olkin Measure of Sampling Adequacy			0.81		
Bartlett's Test of Sphericity			Bartlett's K-squared	df	p-value
			509.95	4	0.000
	PC1	PC2	PC3	PC4	PC5
Standard deviation	1.7013	0.8339	0.7691	0.68033	0.59649
Proportion of Variance	0.5789	0.1391	0.1183	0.09257	0.07116
Cumulative Proportion	0.5789	0.7180	0.8363	0.92884	1.00000
**Energy conservation**					
Kaiser-Meyer-Olkin Measure of Sampling Adequacy			0.67		
Bartlett's Test of Sphericity			Bartlett's K-squared	df	p-value
			464.45	2	0.000
	PC1	PC2	PC3		
Standard deviation	1.4187	0.7752	0.6217		
Proportion of Variance	0.6709	0.2003	0.1288		
Cumulative Proportion	0.6709	0.8712	1.0000		
